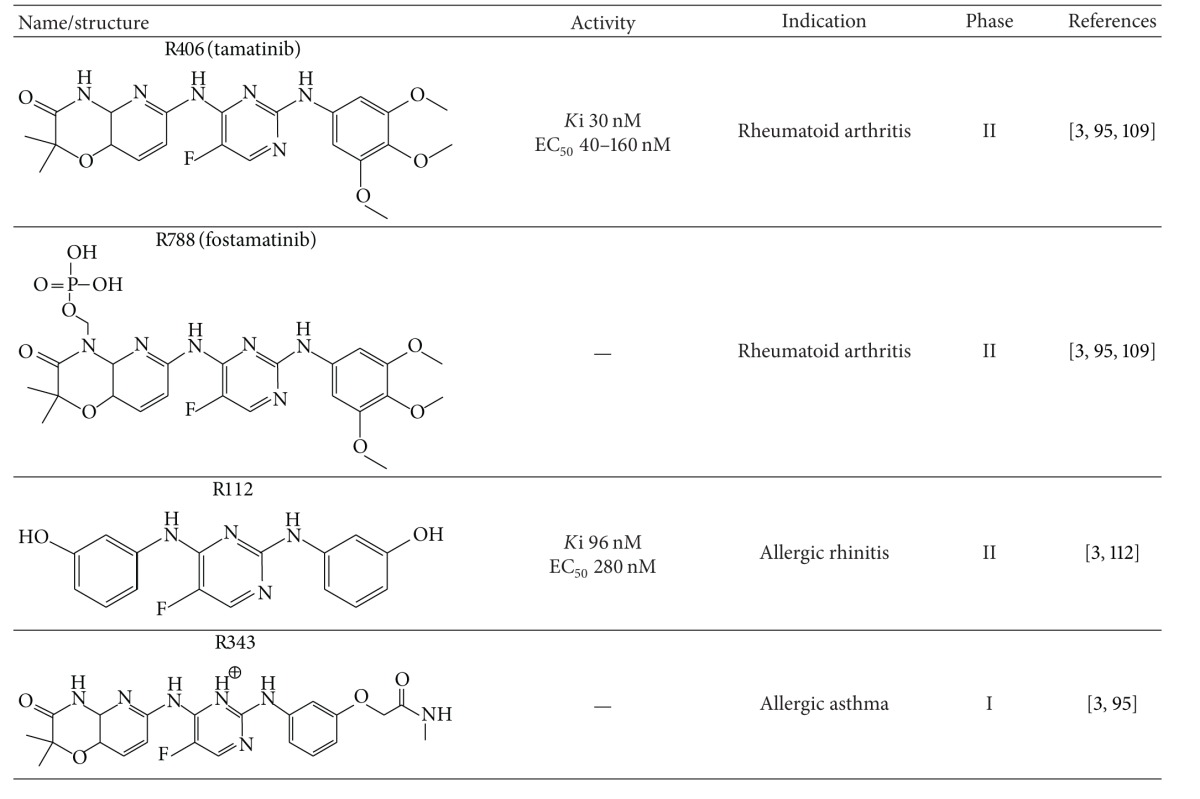# Functional Roles of Syk in Macrophage-Mediated Inflammatory Responses

**DOI:** 10.1155/2014/270302

**Published:** 2014-06-18

**Authors:** Young-Su Yi, Young-Jin Son, Chongsuk Ryou, Gi-Ho Sung, Jong-Hoon Kim, Jae Youl Cho

**Affiliations:** ^1^Department of Genetic Engineering, Sungkyunkwan University, Suwon 440-746, Republic of Korea; ^2^Department of Pharmacy, Sunchon National University, Suncheon 540-742, Republic of Korea; ^3^Department of Pharmacy, College of Pharmacy and Institute of Pharmaceutical Science and Technology, Hanyang University, Ansan, Gyeonggi-do 426-791, Republic of Korea; ^4^Mushroom Research Division, National Institute of Horticultural and Herbal Science, Rural Development Administration, Eumseong 369-873, Republic of Korea; ^5^Department of Veterinary Physiology, College of Veterinary Medicine, Biosafety Research Institute, Chonbuk National University, Jeonju 561-756, Republic of Korea

## Abstract

Inflammation is a series of complex biological responses to protect the host from pathogen invasion. Chronic inflammation is considered a major cause of diseases, such as various types of inflammatory/autoimmune diseases and cancers. Spleen tyrosine kinase (Syk) was initially found to be highly expressed in hematopoietic cells and has been known to play crucial roles in adaptive immune responses. However, recent studies have reported that Syk is also involved in other biological functions, especially in innate immune responses. Although Syk has been extensively studied in adaptive immune responses, numerous studies have recently presented evidence that Syk has critical functions in macrophage-mediated inflammatory responses and is closely related to innate immune response. This review describes the characteristics of Syk-mediated signaling pathways, summarizes the recent findings supporting the crucial roles of Syk in macrophage-mediated inflammatory responses and diseases, and discusses Syk-targeted drug development for the therapy of inflammatory diseases.

## 1. Introduction

Inflammation is the body's first immune response to protect our body from harmful stimuli, such as pathogen infection, damaged cells, and irritants [1, 2]. Symptoms include recruitment of white blood cells, pain, heat, redness, swelling, tissue damages, and dysfunctions of the organs. Inflammation is classified as either acute or chronic. Acute inflammation is immediate immune responses characterized by an increase in the movement of plasma and leukocytes from the blood to the injured sites. Acute inflammation becomes chronic inflammation when the immune system cannot remove invading pathogens or when pathogens continuously invade the body. Chronic inflammation is an inflammation of prolonged duration from several weeks to years, in which active inflammation, tissue injury, and healing occur at the same time. Chronic inflammation is a leading cause of inflammatory/autoimmune diseases, such as rheumatoid arthritis (RA), Alzheimer's diseases, systemic lupus erythematosus (SLE), asthma, psoriasis, atherosclerosis, and cancers [3–6].

Macrophages are white blood cells developed in a myeloid lineage, and differentiated from monocytes in tissues. In inflammation, macrophages have crucial functions, such as antigen presentation, phagocytosis, and immunomodulation via the production of various inflammatory mediators [7, 8]. Macrophages play a critical role in the inflammation that occurs during innate immune responses. Macrophages are activated by the binding with various stimuli, including lipopolysaccharide (LPS), cytokines (e.g., interleukin- (IL-) 1 and tumor necrosis factor- (TNF-) *α*), and other chemical mediators through their receptors (e.g., toll-like receptors (TLRs) and dectin-1 [9, 10]. The binding of stimuli with the receptors on macrophages initiates the stimulatory signals and activates downstream signaling molecules, such as various types of kinases [11–13]. These signals subsequently activate transcription factors, including nuclear factor-*κ*B (NF-*κ*B), activator protein-1 (AP-1), and cAMP response element-binding protein (CREB), resulting in the induction of the expression of proinflammatory genes (e.g., TNF-*α*, COX-2, and iNOS) and the secretion of inflammatory mediators (e.g., nitric oxide (NO), reactive oxygen species (ROS), and prostaglandin E_2_ (PGE_2_)) [14, 15].

Several intracellular signaling molecules are involved and activated during the inflammatory responses in macrophages. Among these signaling molecules, tyrosine kinase families have been considered as the major effector molecule. Spleen tyrosine kinase (Syk) is a nonreceptor tyrosine kinase with a molecular weight of 72 kDa. Syk binds with TLR4 and is activated by phosphorylation mainly at tyrosine residues, resulting in the transduction of stimulatory signals through the activation of various downstream signaling molecules. Since Syk is one of upstream signaling molecules, it orchestrates many downstream signaling molecules and amplifies inflammatory signals. Therefore, Syk has been considered to play critical roles in inflammatory responses.

This review presents a general introduction to Syk as a signaling molecule, discusses the role of Syk in macrophage-mediated inflammatory responses, and summarizes Syk-related inflammatory diseases and Syk inhibitors that could be candidates for the treatment of these diseases. This aim of this review is to increase understanding of the role of Syk in macrophage-mediated inflammatory responses in order to support the development of new therapeutic drugs for the treatment of macrophage-mediated inflammatory diseases.

## 2. Structure and Functions of Syk

### 2.1. Structure of Syk and Its Posttranslational Modifications

**Figure 1:**
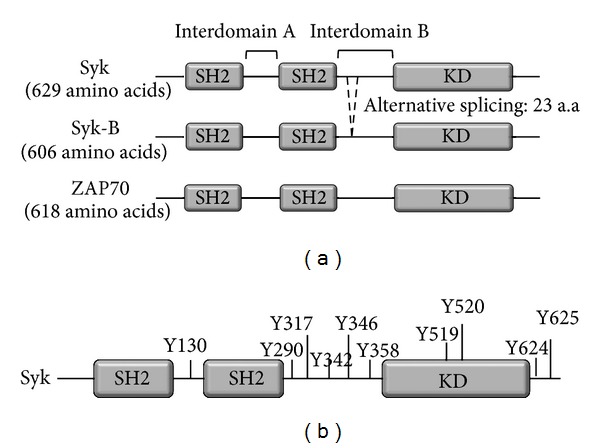
Biochemical features of Syk. (a) Structure comparison of Syk family kinases. (b) Phosphorylation sites of Syk.

Syk is a 72 kDa nonreceptor type of tyrosine kinase consisting of two tandem repeat of Src homology 2 (SH2) domains and one C-terminus kinase domain. These domains are connected with two interdomains: interdomain A between two SH2 domains and interdomain B between C-terminus SH2 domain and a kinase domain (Figure 1(a)) [16–18]. Syk has two forms: the original Syk and an alternatively spliced form called Syk-B that lacks 23 amino acids in interdomain B, including nuclear localization signal peptide (Figure 1(a)) [19, 20]. Although the Syk-B form is found in cells, the exact mechanisms of alternative slicing have not yet been well elucidated. The structure of Syk family kinases among species is highly conserved. Like human Syk, mouse Syk consists of two SH2 domains and a kinase domain at C-terminus [21]. Mouse Syk is only 6 amino acids shorter than human Syk and 92% homologous with human Syk in amino acid sequences. The fruit fly,* Drosophila melanogaster*, has a Syk-related molecule named SH2 domain ankyrin repeat kinase (SHARK), which consist of two SH2 domains and a kinase domain at C-terminus, and unlike Syk, SHARK has ankyrin-like repeats (ANKs) in interdomain A [22]. Hydra has two Syk-like molecules: HTK98 is similar to human Syk, but HTK is more similar to fruit fly Syk, SHARK [23, 24]. Interestingly, the worm* Caenorhabditis elegans* does not have a Syk-like molecule [18]. Mammalian cells express another type of Syk family kinase named zeta-chain-associated protein kinase of 70 kDa (ZAP70) (Figure 1(a)). ZAP70 has almost the same structure as that of Syk, consisting of two SH2 domains and a kinase domain, but shows low homology to Syk in amino acid sequences (less than 50%) and its expression is restricted in certain cell types, such as T cells and NK cells [18]. The characteristics of the Syk family of kinases are summarized in Table 1.

Posttranslational modifications are modifications in the protein synthesis process and are critical steps for the regulation of protein activity. Syk also has posttranslational modifications. Syk is ubiquitinated by Cbl-b and undergoes proteasomal degradation, which negatively regulates B-cell receptor signaling [25]. More importantly, phosphorylation is the most abundant posttranslational modification in Syk. It has been reported that ten sites of Syk are autophosphorylated (Figure 1(b)): one site located in the N-terminal between two SH2 domains, five sites located in the interdomain region between the C-terminal SH2 domain and the kinase domain, two sites located in the kinase domain, and two sites at C-terminus [26]. Syk is phosphorylated on tyrosine residue 317 by Lyn, a Src-phosphotyrosine kinase [27, 28]. Syk is phosphorylated on multiple tyrosine sites, including Tyr317, Tyr342, and Tyr346 in interdomain B under the condition of binding with B-cell receptor [29], and phosphorylated on Tyr630 of human Syk to create binding site for BLNK [30]. Syk is also phosphorylated on serine residues. Phosphorylation of Syk on Ser297 is observed within 5 min after the stimulation of B-cell receptor [31]. Unlike ubiquitination and phosphorylation, other types of posttranslational modifications of Syk, such as methylation and acetylation, have not yet been clearly reported.

### 2.2. Localization and Tissue Specificity of Syk

Syk localizes mostly in cytoplasm [32]. As previously reported, Syk interacts with a number of proteins mainly located in cytoplasm or plasma membrane, implying that the fraction where Syk is mainly located is cytoplasm. However, Syk is also found in the nucleus [33]. Syk was reported to be translocated from nucleus to plasma membrane to interact with B-cell receptor under the activation of B-cell receptor [33]. Although Syk is found in both nucleus and cytoplasm, the exact molecular mechanisms that trigger the translocation of Syk from nucleus to cytoplasm or in the reverse direction have not yet been well elucidated.

Following an initial report of being highly expressed in hematopoietic cells [34], Syk was thought to be a hematopoietic cell-specific kinase. Later studies, however, reported that Syk is widely expressed in nonhematopoietic cells, including epithelial cells, fibroblasts, and neuronal and vascular endothelial cells [34, 35]. Interestingly, Syk is highly expressed in peritoneal macrophages and Raw 264.7 cells, a murine macrophage-like cell line. Under the stimulation of cells by various TLR ligands, such as LPS, poly I:C, and Pam3CSK4, Syk is highly activated within several minutes [36], implying that it plays a pivotal role in macrophage-mediated innate immunity through TLRs.

### 2.3. Syk-Binding Proteins

Identification of Syk-binding proteins is critical because activation of Syk is induced by a number of physiological ligands. Along with the activation of Syk by autophosphorylation, Syk is also activated by phosphorylation through the binding with upstream kinases and activated Syk binds with downstream substrate proteins to transduce its signaling. Proteome analysis using amine-specific isotope tag and GFP nanotrap identified Syk-binding proteins with high efficiency and affinity, such as CrkL, BLNK, tubulins, Csk, RanBP5, and DJ-1 [37]. Several upstream kinases have been identified to bind with and then activate Syk. Lyn binds and phosphorylates with Syk [27]. RhoH, an atypical G protein, binds with Syk and is followed by Syk activation [38]. Nuclear factor of activated T cells- (NFAT-) activating molecule 1 (NFAM1), which is an ITAM-containing cell surface molecule, also binds with Syk through its phosphorylated ITAM motif [39]. TLR binds with Syk and regulates signaling [40]. Syk also binds with the following of its downstream proteins: (1) adapter proteins, such as linker for activation of T cells (LAT) [41], SH2 domain-containing leukocyte protein of 76 kDa (SLP-76) [42], B-cell linker protein (BLNK) [43], B-cell adaptor for PI3K (BCAP) [44], HS-1 (also known as SPY75) [45], Src homology and collagen (SHC) [46], and T-cell receptor CD3*ζ* (TCR CD3*ζ* [47] and 3BP2 [48]); (2) enzymes, such as Cb1 [49], phospholipase C-*γ* (PLC-*γ*) [50], phospholipase D (PLD) [51], Bruton's tyrosine kinase (BTK) [52], proline-rich tyrosine kinase 2 (PYK2) [53], Vav1 [54], phosphatidylinositol 3 kinase (PI3K) [55], mitogen-activated protein kinases (MAPK), extracellular signal-regulated kinase (ERK), and janus kinase (JNK) [56–58]; and (3) cytoskeletal components and cell adhesion molecules, such as tubulin [59], SH3 domain-containing protein 7 (SH3P7) [60], and *β*-integrins [61, 62].

### 2.4. Roles of Syk in Signal Transduction

Syk plays a critical role in immunoreceptor signaling pathways. Many immunoreceptors, including B-cell receptor, T-cell receptor, and various FcRs, contain immunoreceptor tyrosine-based activation motifs (ITAMs) in their cytoplasmic domains, and the ITAMs are phosphorylated by Src-family kinases. The first step of Syk activation is its binding to phosphorylated ITAMs of the immunoreceptors. Once ITAMs are phosphorylated by Src-family kinases, Syk is recruited to the phosphorylated ITAMs and binds with phosphorylated tyrosine residues in ITAMs through its SH2 domains. This binding leads to conformational change and unfolding of Syk, resulting in consequent activation of Syk through the autophosphorylation itself as well as the phosphorylation by other upstream kinases, such as Lyn [27]. The activated Syk which gains an enzymatic activity transduces its signaling cascades to downstream through activating its downstream substrate proteins by phosphorylating them and sometimes even directly interacting them. Therefore, most critical events for initiating Syk activation are the phosphorylation of ITAMs in immunoreceptors and subsequent binding of Syk to the phosphorylated ITAMs through its SH2 domains. As described in Section 2.3, Syk binds with a variety of upstream signaling molecules and its downstream substrates, and these interactions activate Syk-mediated signaling pathways.

Syk is involved in cell adhesion molecule signaling.* In vitro* and* in vivo* studies show that Syk is activated in integrin signaling transduction, resulting in the increase in adhesion of leukocytes to the inflamed endothelial cells [63–65]. Syk also interacts with P-selectin glycoprotein ligand 1 (PSGL1) through ITAM motifs of PSGL1. With the interaction of Syk with PSGL1, Syk is subsequently activated in the PSGL1 signal transduction pathway in leukocytes [66, 67], and inhibition of Syk activity results in the suppression of PSGL1-mediated transcriptional regulation [66].

Syk shows its roles in innate pathogen recognition. Recently, several studies have reported that Syk is a key molecule in the signaling pathways initiated by pattern recognition receptors (PRRs) in terms of recognizing pathogen-associated molecule patterns (PAMPs) and activating innate immune responses mediated by Syk is activated through the interaction with C-type lectin CLEC7A, which is a mammalian PRR for fungal *β*-glucans [68], and this Syk-CLEC7A interaction stimulates its signaling pathways, resulting in the production of ROS [69, 70] and phagocytosis of yeast by dendritic cells [68]. Syk is also activated in the signaling pathways triggered by the recognition of bacteria and viruses. The binding of mycobacterial PAMPs to their receptors, CLEC7A, CLEC6A, or CLEC4E, stimulates Syk activation in their signal transduction [71], and Syk knock-out mice show attenuated antibacterial host defense [72]. The binding of dengue virus to its receptor, CLEC5A, activates Syk and induces the production of proinflammatory cytokines through the binding of Syk with phosphorylated DAP12, which is an adaptor molecule between CLEC5A and Syk [73].

Syk is also activated through nonpathogenic stimuli, such as the molecules produced from damaged cells. The ligands produced by necrotic cells bind to their receptors, such as CLEC4E associated with Fc*γ*R or CLEC9A, resulting in the recruitment and activation of Syk [74–76]. Interestingly, Syk is involved in the signaling of the Fc*γ*R-mediated, antibody-dependent cellular cytotoxicity (ADCC) reaction. The Fc*γ*R-mediated ADCC reaction is suppressed in Syk and ZAP70-defective natural killer cells, implying that Syk plays a critical role for a Fc*γ*R-mediated ADCC reaction [77].

## 3. Functions of Syk in Macrophage-Mediated Inflammatory Responses and Diseases

Although the functions of Syk have been intensively studied in adaptive immune responses, especially in B-cell signaling, its functions in other types of cells, such as macrophages, have also been studied. This section examines the functions of Syk in macrophage-mediated inflammatory responses.

Macrophages function as the first line of defense against invading pathogens. Macrophages use cell surface receptors to detect the pathogens, for example, PRRs such as TLRs, to initiate signaling inside the cells, and then to activate appropriate immune responses through its cytoplasmic domain. One of the most critical molecules in innate immune responses is intracellular nonreceptor tyrosine kinases, such as Syk, which is the only Syk family kinase found in innate cells.

**Figure 2:**
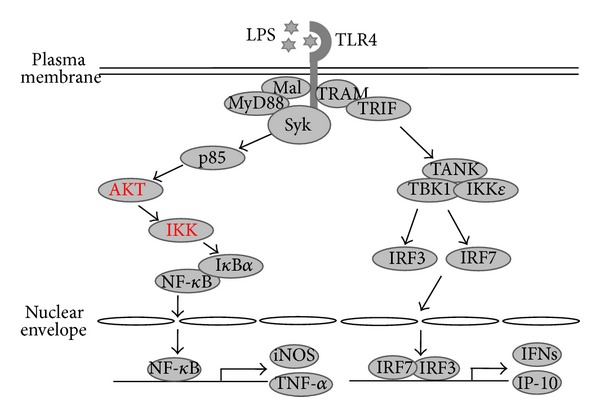
Syk-regulated signaling pathways in macrophage-mediated inflammatory responses.

Syk is involved in TLR4 signaling (Figure 2). Syk is recruited to TLR4 in macrophages [78] and binds with TLR4 through its N-terminal SH2 domain [79]. Syk is activated in macrophages under LPS stimulation. Syk is very quickly activated (within 2 min) by phosphorylation under LPS stimulation condition. Syk activation leads to the subsequent activation of downstream signaling molecules, such as p85, AKT, IKK, PDK1, and NF-*κ*B, resulting in the induction of expression of proinflammatory genes, such as TNF-*α*, COX-2, and iNOS, and the production of inflammatory mediators, such as NO and PGE_2_ in macrophages [80–83]. Syk is also activated by minimally oxidized low-density lipoprotein (mmLDL). Once mmLDL binds to TLR4, Syk binds with LTR4 and is activated, and subsequently activates Vav1, PLC-*γ*, and JNK, resulting in cytoskeleton rearrangement, ROS generation, and cytokine secretion in macrophages [84]. Since Syk activation induces inflammatory responses in macrophages, a number of previous studies have shown that Syk inhibition suppresses inflammatory responses in macrophages. A variety of plant extracts has been reported to suppress inflammatory responses through the inhibition of Syk activity. The ethanol extract of* Cinnamomum cassia* suppresses the production of NO, PGE_2_, and TNF-*α* in LPS-stimulated macrophages [83]. The methanol extract of* Evodia lepta *suppresses the production of NO and PGE_2_ in LPS-stimulated macrophages and ameliorates the symptoms of EtOH/HCl-induced gastritis [80]. The ethanol extract of* Artemisia asiatica*, the methanol extract of* Cerbera manghas, *and the methanol extract of* Hopea odorata *also suppress the production of NO, PGE_2_, and TNF-*α* in LPS-stimulated macrophages and ameliorate the symptoms of EtOH/HCl-induced gastritis [85–87]. The methanol extract of* Archidendron clypearia* suppresses the production of NO, PGE_2_, and TNF-*α* in LPS-stimulated macrophages and ameliorates the symptoms of dextran sodium sulfate (DSS)-induced colitis [82]. The ethanol extract of* Myrsine seguinii* also suppresses the production of NO, PGE2, and TNF-*α* in LPS-stimulated macrophages and ameliorates the symptoms of thioglycollate-induced peritonitis [88]. The methanol extract of* Rhodomyrtus tomentosa* suppresses the production of NO and PGE2 in LPS-stimulated macrophages and ameliorates the symptoms of EtOH/HCl-induced gastritis and DSS-induced colitis [81]. Single compounds or chemicals also suppress inflammatory responses through the inhibition of Syk activity. Piceatannol, a pharmacological inhibitor of Syk, strongly suppresses inflammatory responses [36], and caffeic acid, which inhibits Syk activation, suppresses inflammatory responses in macrophages [89]. Quercetin, a major bioflavonoid present in fruits and vegetables, also inhibits Syk activation, resulting in the suppression of inflammatory responses in macrophages [90]. The plant extracts and natural compounds that suppress macrophage-mediated inflammatory responses through the inhibition of Syk are summarized in Table 2. On the contrary to the majority of previous studies that Syk activation induces inflammatory responses in macrophages, Syk was also reported to have a suppressive role in inflammatory responses. The secretion of proinflammatory cytokines, such as TNF, IL-6, and IL-12 was dramatically increased in Syk-deficient bone marrow-derived macrophages compared to wild type macrophages in response to various TLR ligands, including LPS, CpG DNA, and lipopeptide [91]. These results were supported by another result that the secretion of above proinflammatory cytokines was also increased in the bone marrow-derived macrophages lacking DAP12 which is an upstream signal cascade molecule of Syk compared to wild type macrophages in the response of TLR ligands [91]. Although this observation is contradictory to a lot of previous studies, it might be possible in the aspects that the bone marrow-derived macrophages used in this study were generated from Syk-deficient fetal liver chimeras because Syk-deficient mice are embryonic lethal [92]; therefore, the function of Syk in these macrophages might be different from that of conventional effector macrophages in inflammatory lesions. Moreover, the doses of TLR ligands used in this study to stimulate bone marrow-derived macrophages seem relatively low, whereas the doses in other studies which showed a stimulatory role of Syk in inflammatory responses in RAW264.7 cells and peritoneal macrophages were relatively high and widely used levels; in addition, the secretion of the proinflammatory cytokines did not show clear dose-dependency with TLR ligands, implying that the role of Syk in macrophage-mediated inflammatory response might be also different depending on the doses of TLR stimulatory ligands. Taken together, these findings strongly support the crucial role played by Syk in macrophage-mediated inflammatory responses.

## 4. Syk-Targeted Drug Development

### 4.1. Syk Inhibitors

Since Syk plays a crucial role in B-cell activation and in innate immune cell-mediated immune responses, especially macrophage-mediated inflammatory responses which subsequently contribute to the pathogenesis of various diseases, several Syk inhibitors have been actively developed, some of which are currently in clinical trials to treat human diseases. This section examines the Syk inhibitors that have been developed and are under development.

Most Syk inhibitors are synthetic derivatives. Various types of nuclei, such as naphthyridines, pyrimidine-5-carboxamide, imidazole[1,2-c]pyrimidine, 1,2,4-triazolo[4,3-c]pyrimidine, 4-thiazolyl-2-phenylaminopyrimidines, and oxindoles, have been modified to increase their inhibitory potencies against Syk. Naphthyridine is a basic nucleus that shows Syk inhibitory activity. Seventeen derivatives of this compound have been developed to increase the inhibitory potency, and their IC_50_ values range from 90 *μ*M to several nM [3]. Pyrimidine-5-carboxamide shows Syk-specific inhibitory potency. Eleven derivatives of pyrimidine-5-carboxamide have been synthesized with IC_50_ values ranging from over 10 *μ*M to 23 nM [3]. Imidazole[1,2-c]pyrimidine has seven derivatives with IC_50_ values ranging from over 7.1 *μ*M to 6 nM, and 1,2,4-triazolo[4,3-c]pyrimidine has six derivatives with IC_50_ values ranging from over 7.7 *μ*M to 4 nM [3]. Six derivatives of 4-thiazolyl-2-phenylaminopyrimidines have been developed with IC_50_ values ranging from 1.8 *μ*M to 4 nM, and five derivatives of oxindoles have been developed with IC_50_ values ranging from 8.1 *μ*M to 5 nM [3].

Natural Syk inhibitors, such as curcumin and piceatannol, have also been reported. Curcumin inhibits Syk signaling pathways and could be used in the treatment of B-cell lymphoma [93]. Piceatannol also inhibits Syk activity, but its inhibitory action has not yet been well elucidated [94]. Several Syk inhibitors are currently in clinical trials (Table 3). R406, R788, R112, and R343 are structurally related analogues that are selective ATP-competitive Syk inhibitors; they were developed to treat a variety of inflammatory and allergic diseases [3, 95]. BAY-61-3606, identified by Yamamoto et al., is an imidazopyrimidine analogue that selectively inhibits Syk activity to treat allergic diseases [96]. Pyrimidine-5-carboxamides families, such as YM193306 and other derivatives developed by Hisamichi et al. are also Syk inhibitors to treat allergic diseases [97].

### 4.2. Syk in Inflammatory Diseases and Syk-Targeted Therapeutics

Many studies using* in vivo* animal models and Syk knock-out experiments have demonstrated that Syk is involved in various inflammatory diseases, such as RA, allergic asthma/rhinitis, intestinal ischemia reperfusion injury, SLE, and idiopathic thrombocytopenic purpura (ITP), and that Syk inhibition could be a therapeutic strategy for the treatment of these inflammatory diseases [98–103]. Syk is expressed and activated in the synovium of RA [104]. Activated Syk increases TNF-*α*-induced cytokine expression through the suppression of JNK signaling in fibroblast-like synoviocytes [104]. Moreover, several studies have revealed that Syk plays a pathological role in animal models of arthritis [105–107]. An orally available drug, R406, and R788, a prodrug of R406, show antiarthritic effects in the collagen-induce arthritis animal model [105], and immunotherapy treatment of RA targeting activated macrophages where Syk is activated ameliorates the arthritic symptoms in an animal model [108]. Moreover, these two drugs are in clinical trial phase II for the treatment of RA and show significant effects in the patients with active RA [109]. Syk is involved in allergic conditions, such as allergic rhinitis and asthma, through the activation of IgE production. Syk is critical for activating the mediators of degranulation, eicosanoid, and cytokine production [110]. R112 inhibits IgE-mediated histamine release from human basophils [111], and its intranasal administration significantly decreases the clinical symptoms of allergic rhinitis in clinical trial phase II [112]. R343 developed most recently is in clinical trial phase I with an inhaled formulation for the treatment of allergic asthma [95]. Hematopoietic cells are involved in the expression of ischemia reperfusion injury, implying that targeting Syk could be applied to the treatment of ischemia reperfusion injury [113]. R788 has been reported to suppress the symptoms of ischemia reperfusion injury in an animal model [113, 114]. Syk is also involved in the pathogenesis of SLE. The pathogenesis of SLE correlates to B-cell activation where Syk could play a critical role [115]. The symptoms of SLE are ameliorated by the R788 in lupus-prone MRL/lps and BAX/BAK mice [116, 117]. ITP is mediated by the production of IgG specific for antigens on platelets. Since Syk is involved in FcgR-mediated signaling activation, it may participate in this disease. Indeed, the Syk inhibitors R406 and R788 ameliorated the symptoms of ITP in an animal model [102] and in human clinical trials. In addition to these inflammatory diseases, Syk may play a critical role in activated macrophage-mediated inflammatory diseases, including Crohn's disease, psoriasis, and atherosclerosis, since Syk is a critical kinase for macrophage-mediated inflammatory responses. Another orally administrable, selective Syk inhibitor, BAY-61-3606, exhibits an inhibitory effect of degranulation and cytokine synthesis in mast cells and suppresses the symptoms of antigen-induced airway inflammation, including asthma and rhinitis, in* in vivo* animal models [96]. However, Bay-61-6303 exhibits its target selectivity profile limiting to only 6 kinases and shows significant off-target effects [118], implying that BAY-61-6306 may not be a good candidate for clinical trials and drug development.

## 5. Conclusions and Perspective

Although inflammation is the process of protecting our body from invading pathogens, excessive and chronic inflammatory responses are major causes of various types of diseases, including inflammatory/autoimmune diseases and cancers. Increasing evidence has revealed that activated macrophages are major effector cells in inflammatory responses and inflammatory diseases. Several studies have demonstrated that Syk is critically involved in macrophage-mediated inflammatory responses and in inflammatory diseases, including RA, allergic asthma/rhinitis, SLE, intestinal ischemia reperfusion injury, and ITP. This implies that Syk is a crucial player in inflammatory responses and could be a potent target to treat inflammatory diseases. Although many Syk inhibitors have been and are being developed, most suffer shortcomings such as cytotoxicity, low therapeutic efficacies, and off-target effects due to multiple kinase targeting. This necessitates the development of new potential Syk inhibitors with properties that offer both lower cytotoxicity and higher therapeutic efficacy. We expect novel Syk inhibitors, especially novel biological drugs that selectively target Syk, with strong anti-inflammatory effects and minimal toxicity, to be developed for the treatment of macrophage-mediated inflammatory diseases in the near future.

## Figures and Tables

**Table 1:** Syk family kinases and their characteristics.

Family	Domains	Expressing cells	Functions	References
Syk	Two SH One kinase	Hematopoietic cells	Cell adhesion	[18, 36, 80–83, 85–90]
		B cells	Innate pathogen recognition	
		Macrophages	Tissue damage recognition	
		Mast cells	Inflammatory responses	
		Neutrophils	Antibody-dependent cellular cytotoxicity	

Syk-B	Two SH	Lymphocytes	Interference with Syk biological activities	[18, 20]
	One kinase	Breast tumor cells		

ZAP70	Two SH	T cells	T-cell development and activation	[18, 119, 120]
	One kinase	Natural killer cells	Antibody-dependent cellular cytotoxicity	

**Table 2:** Summary of the plant extracts and naturally occurring compounds targeting Syk.

Plant name	Activity	References
Plant extracts		
*Evodia lepta *	Suppression of NO, PGE_2_ production Amelioration of EtOH/HCl-induced gastritis	[80]
*Rhodomyrtus tomentosa *	Suppression of NO, PGE_2_ production Amelioration of EtOH/HCl-induced gastritis and DSS-induced colitis	[81]
*Cerbera manghas *	Suppression of NO production Amelioration of EtOH/HCl-induced gastritis	[121]
*Archidendron clypearia *	Suppression of NO, PGE_2_, TNF-*α* production Amelioration of DSS-induced colitis	[82]
*Cinnamomum cassia *	Suppression of NO, PGE_2_, TNF-*α* production	[83]
*Artemisia asiatica *	Suppression of NO, PGE_2_, TNF-*α* production Amelioration of EtOH/HCl-induced gastritis	[122]
*Hopea odorata *	Suppression of NO, PGE_2_, TNF-*α* production Amelioration of EtOH/HCl-induced gastritis	[87]
*Myrsine seguinii*	Suppression of NO, PGE_2_, TNF-*α* production Amelioration of thioglycollate-induced peritonitis	[88]

Natural compounds		
Piceatannol	Suppression of NO, PGE_2_, TNF-*α* production	[36]
Caffeic acid	Suppression of NO, PGE_2_, TNF-*α* production Amelioration of EtOH/HCl-induced gastritis	[89]
Quercetin	Suppression of NO, PGE_2_, TNF-*α*, IL-1*β*, IL-6, GM-CSF production	[90]

**Table 3:** Summary of Syk inhibitors in clinical trials.